# [Retracted] circRNA_PTPRA functions as a sponge of miR-582-3p to regulate hepatocellular carcinoma cell proliferation, migration, invasion and apoptosis

**DOI:** 10.3892/etm.2025.13033

**Published:** 2025-12-01

**Authors:** Yanlin Jin, Yi Zhang, Xiaoming Luo

Exp Ther Med 22:1276, 2021; DOI: 10.3892/etm.2021.10711

Following the publication of this paper, it was drawn to the Editor’s attention by a concerned reader that flow cytometric (FCM) assay data featured in Figs. 3A and 6A were strikingly similar to FCM data which were ultimately published in a number of other papers in different journals that were written by different authors at different research institutes; moreover, comparing the data for the cell migration and invasion assay experiments shown in Figs. 2C, D and 5C revealed the presence of two pairs of overlapping data panels, where the data were intended to show the results of differently performed experiments, and the histograms shown in Fig 4 were also markedly similar to the dimensions of histograms that were featured in the same papers of concern (including the style of design of the bar charts).

Owing to the fact that the contentious data in the above article were found to be strikingly similar to data that have appeared elsewhere in other papers in the scientific literature, the Editor of *Experimental and Therapeutic Medicine* has decided that this paper should be retracted from the Journal. The authors were asked for an explanation to account for these concerns, but the Editorial Office did not receive a reply. The Editor apologizes to the readership for any inconvenience caused.

